# One and a half years into the COVID-19 pandemic - exit strategies and efficacy of SARS-CoV-2 vaccines for holistic management and achieving global control

**DOI:** 10.3906/sag-2106-236

**Published:** 2021-12-17

**Authors:** Eskild PETERSEN, Deniz GÖKENGİN, Asma Al BALUSHI, Alimuddin ZUMLA

**Affiliations:** 1 European Society for Clinical Microbiology and Infectious Diseases, Emerging Infections Task Force, ESCMID, Basel Switzerland; 2 Institute for Clinical Medicine, Aarhus University Denmark; 3 Depatmernt of Infectious Diseases and Clinical Microbiology, Faculty of Medicine, Ege University, İzmir Turkey; 4 Department of Infectious Diseases Department, Internal Medicine, Sohar Hospital Oman; 5 Division of Infection and Immunity, Center for Clinical Microbiology, University College London and NIHR Biomedical Research Centre, UCL Hospitals NHS Foundation Trust, London United Kingdom

**Keywords:** COVID-19, Vaccines, surveillance, zoonosis, variants

## Abstract

One and a half years into the pandemic, SARS-CoV-2 is still here to stay. Whilst rapid several effective COVID-19 vaccines have been developed and are being rolled out, the critical questions remain whether vaccines provide widespread protection against infection and reinfection, and what the duration of protection is. Community wide control cannot be obtained until almost everyone is immune. Vaccine production must be ramped up to cover the world population. The price of herd immunity through natural infection is high mortality in the elderly and morbidity in other age groups including children and Long-COVID. We must expect a new wave in the coming winter. The severity will depend on the proportion of the population with immunity from natural infections or immunisation. Therefore, control rests on a population wide immunisation including children, which may or may not need to be repeated if new SARS-CoV-2 variants evolve that can escape immunity from either previous infections or immunisations. Preventing long term sequelae of COVID-19 also remains a priority.

## 1. Introduction

When COVID-19 pandemic was declared a global emergency, in early 2020, the scientific and public health communities hypothesized that SARS-CoV-2 transmission would eventually be brought under control by herd immunity, conferred by natural infection, vaccine development and rollout, or both [1]. Whilst rapid several effective COVID-19 vaccines have been developed and are being rolled out, the critical questions remain whether vaccines provide widespread protection against infection and reinfection, and what the duration of protection is [2].

One and a half years ago when the SARS-CoV-2 outbreak started in China, most experts expected a course like pandemic influenza with seasonal transmission for three to four months, followed by a pause over the summer and a second wave next winter.

SARS-CoV-2 has proved very different with sustained transmission during all seasons and epidemic waves have only been brought under control by severe, country-wide interventions to prevent transmission, strict movement and travel restrictions, so called lock-downs associated with implementation of infection control measures. With massive rollout of COVID-19 vaccines, most high-income countries have protected the most vulnerable, and are heading towards relaxing lockdown restrictions, although low-income countries have not benefitted yet. The inequalities in vaccine distribution and access need to be addressed seriously if global COVID-19 control is to be achieved.

## 2. Characteristics and implications of the pandemic

### 2.1. SARS-CoV-2 versus influenza

So why does SARS-CoV-2 behave different from pandemic influenza? One hypothesis could be that we have all been exposed to influenza throughout life by either natural infections or immunisations, which has created a population background immunity. In contrast, humans had no background immunity against SARS-CoV-2 since it is a novel zoonotic pathogen. Therefore, repeated waves of SARS-CoV-2 coupled with worldwide immunisation campaigns are required to build herd immunity. The trajectory of spread of novel viruses is variable, with some novel viruses becoming less virulent with time or disappearing altogether. For SARS-CoV-2 we do not know.

### 2.2. Transmissibility of SARS-CoV-2 – the basic reproduction number, R0

Knowledge of the basic reproductive number R_0_ ****over time is important for understanding the dynamics of SARS-CoV-2 transmission. Most estimates indicate an R_0_ around 2.5 with a doubling time of around 5 days. Excretion of the virus is at its peak when symptoms start which severely reduces the effect of quarantining symptomatic people. Besides, a proportion of infected people are asymptomatic [3,4]. There remain several knowledge gaps regarding how transmission of COVID-19 is ongoing in different geographical settings across the world.

### 2.3. Herd immunity and immunisation of children

Herd immunity, defined as a “high proportion of immunity in a population that prevents further transmission” has proven elusive for SARS-CoV-2 transmission during the first 18 months of the pandemic. With a R_0_ value of approximately 2.5, transmission would need to be reduced by more than 60% (1–1/R_0_) to reach a R_e_ (basic reproduction number after mitigation efforts) of less than 1. If the R_0_ is higher as it has been implicated with some of the variant SARS-CoV-2 lineages, so called variants of concern (VOC). World Health Organization (2021). WHO announces simple, easy-to-say labels for SARS-CoV-2 Variants of Interest and Concern [online]. Website https://www.who.int/news/item/31-05-2021-who-announces-simple-easy-to-say-labels-for-sars-cov-2-variants-of-interest-and-concern [accessed 06 June 2021]. A larger proportion of the population needs to be immune. That means that we have to immunize a high proportion of the population probably up towards 90%. 

That will not be possible without immunizing children perhaps down to the age of two years and perhaps in the future a vaccine against SARS-CoV-2 will be included in the childhood immunisation program [5]. Children can also be severely ill from SARS-CoV-2. A study from France [6] found that among patients younger than 18 years, the rates of intensive care unit (ICU) admission were significantly higher for COVID-19 than for influenza. The need for intensive care was highest in patients with COVID-19 who were younger than 5 years, 2.3% for COVID-19 versus 0.9% for influenza. Mortality was ten-times higher in children aged 11–17 years admitted to hospital with COVID-19 than in patients in the same age group admitted with influenza, 1.1% versus 0.1% [6]. COVID-19 is not an innocent infection in children and adolescents.

VOCs might be able to evade infection or vaccine-induced neutralizing antibodies, thus lead to a growing number of reinfections. One study demonstrated that cross reactivity of SARS-CoV-2 specific T-cell mediated immunity might protect against severe disease [7]. 

In addition, a recent study in Israel where a large proportion of population have been vaccinated showed that there is a cross protection of unvaccinated individuals, hence a significant reduction in transmission rates [8]. The duration of protection is not yet well known and further studies are required.

Thus, a strategic plan to ensure a faster and widespread immunization of the population including children is needed.

### 2.4. Transmission of SARS-CoV-2 variants and future modelling

An early modelling (May 2020) of different scenarios predicted recurrent waves depending on acquired population immunity and R_0 _[9]. The model did not account for emergence of new variants or vaccines. A later study predicted that “once the endemic phase is reached and primary exposure is in childhood, CoV-2 may be no more virulent than the common cold” [10]. However, this presumptive benign nature of SARS-CoV-2 and especially the VOCs can be questioned. Protective immunity against VOCs either from infections with previous lineages or vaccines are not known, but an influenza like situation where acquired immunity is not fully protective can easily be imagined. 

Early studies have shown that PfizerBioNTech and Moderna mRNA COVID-19 vaccines were highly effective to prevent SARS-CoV-2 infection during the period when the majority of infections are believed to have been the original SARS-CoV-2 not yet being replaced by the alpha variant [11–13]. High protective efficacy (83.5% and 65.9%, respectively) was also reported for an inactive virus vaccine CoronaVac [Sinovac Life Sciences (Beijing, China)] in two different studies from Turkey and Chile; considering the time periods of both studies, the majority of infections in the studies are thought to have been the original SARS-CoV-2 and the alpha variant, respectively [14,15]. A more recent study that examined virologic efficacy of three [Pfizer-BioNTech, Moderna, and Janssen (Johnson & Johnson)] COVID-19 vaccines before and during the predominance of SARS-CoV-2 B.1.617.2 (Delta) variant reported that the adjusted efficacy was 66% (95% CI = 26%–84%) during the Delta-predominant period versus 91% (95% CI = 81%–96%) during the pre-Delta period. (16) We are not sure whether this loss of efficacy is due to the low efficacy of the vaccine against the delta variant or to the declining protective effect of the vaccine within time. No study exists on the efficacy of inactive coronavirus vaccines on VOCs.

In Table 1, we have attempted to compare what we know about major VOCs and vaccine protective efficacy. The numbers are approximate but illustrate that the protective efficacy of the different vaccines is different and more studies are needed especially monitoring COVID-19 in populations after infections and immunisations and extensive sequencing of new infections become more and more important as the virus evolves. 

**Table 1:** Approximate protective efficacy of major SARS-C0V-2 vaccines against the major antigenic variants, so called variants of concern(VOC).

VOC new nomen-clature	Previousnomen-clature	Country first identified	Protective efficacy				
			Pfizer/Biontech;Moderna	Astra/Zeneca	Johnson&Johnson	Novavax	Sinovac
a	B.1.1.1.7	United Kingdom	100%	75% (66%)	75%	90%	50.7%–83.5%
b	B.1.351	South Africa	75%	20%	60%	50%	Not known
g	P.1	Brazil	Not known	Not known	70%	Not known	Not known
d	B1.617.2	India	88%	60%–65%	Not known	80%	Not known

### 2.5. Vaccine supply

The World Health Organization (WHO) Strategic Advisory Group of Experts on Immunization (SAGE) released a document on September 14, 2020 to promote equitable global access to safe and effective vaccines among all countries, giving emphasis to low-and middle-income countries. World Health Organization (2021). WHO SAGE values framework for the allocation and prioritization of COVID-19 vaccination [online]. Website https://apps.who.int/iris/bitstream/handle/10665/334299/WHO-2019-nCoV-SAGE_Framework-Allocation_and_prioritization-2020.1-eng.pdf?ua=1 [accessed 10 June 2021]. However, current vaccination rates range from 13% in India where the burden of the pandemic is huge and the consequences are devastating, to 63% in Israel and only 39.4% of the global population received at least one dose. The situation is far worse in low-income countries with only 1.7% of people having received at least one dose. Our World in Data (2021). Coronavirus (COVID-19) Vaccinations [online]. Website https://ourworldindata.org/covid-vaccinations [accessed 10 June 2021]. The limited production capacity, shortage of raw materials and consumables used in the production process coupled with the purchase agreements signed by high-income countries in advance have already hampered access to vaccines by less developed and lower-income countries creating an inequity between regions and populations similar to that experienced in 2009 with the influenza vaccine [17–19]. Many countries do not seem to be likely to achieve massive vaccination until the end of 2022, which creates a major challenge in controlling the epidemic.

### 2.6. Long term sequelae “Long-COVID”

One recent study found that SARS-CoV-2 coursed substantial immune activation in the central nervous system with pronounced neuropathology [20]. Altered brain T cell–microglial interactions were linked to clinical measures of systemic inflammation and disturbed hemostasis [21]. Recent data from the United Kingdom shows that twelve weeks after acute COVID-19, 13.7% continued to experience symptoms. Office for National Statistics (2021). Prevalence of ongoing symptoms following coronavirus (COVID-19) infection in the UK: 4 June 2021. Statistical Bulletin, Office for national Statistics, United Kingdom, 2 May 2021 [online]. Website https://www.ons.gov.uk/peoplepopulationandcommunity/healthandsocialcare/conditionsanddiseases/bulletins/prevalenceofongoingsymptomsfollowingcoronaviruscovid19infectionintheuk/4june2021 [accessed 10 June 2021]. Among people suffering from Long-COVID 63.7% experienced some limitation to their day-to-day activities and 18.8% reported that their day-to-day activities had been limited a lot.^4^

The number of people with Long-COVID in a country will thus depend on the proportion obtaining immunity from natural infection compared to immunization. A proportion of people with Long-COVID will seek medical help and thus there will be a pressure on the health care systems to investigate and help these patients. Probably special Long-COVID outpatient clinics and rehabilitation programs need to be developed and we do not know what happens in the long term after twelve weeks. Therefore, there will be an increased burden on health care systems even months after the outbreak peaked.

### 2.7. The zoonotic connection

Modelling studies can give a certain prediction but cannot predict the future. The outbreak in India was most probably started by the mass gathering Hindu festival Kumbh Mela and this illustrates how quickly the situation can spin out of control and how public health precautions need to continue for the foreseeable future.

The SARS-CoV-2 is a zoonosis probably arising among farmed animals in China. The outbreak in mink and the easy transmission between humans and mink was a warning [21,22]. We need to intensify surveillance of SARS-CoV-2 and other corona virus in animals, especially farmed animals living in cramped conditions and in close contact to humans. There has been limited reports of infections in dogs and felines but coronavirus may in theory infect any mammal. 

A recent study found that between May 2017 and November 2019, 38 different animal species of which 31 were protected were sold at Wuhan markets [23]. It is a real threat that SARS-CoV-2 may disappear into some animal reservoir and reenter humans later depending on the genetic evolution in that species and the population immunity in human populations just as for influenza [24]. Therefore, enhanced surveillance of farmed animals especially in large, indoor facilities (pigs, poultry, cattle, etc.) including sequencing to identify SARS-CoV-2 variants is urgently needed.

## 3. Conclusion

SARS-CoV-2 is here to stay. Community wide control cannot be obtained until almost everyone is immune. Vaccine production must be ramped up to cover the world population. 

The price of herd immunity through natural infection is high mortality in the elderly and morbidity in other age groups including children and Long-COVID. 

We must expect a new wave in the coming winter. The severity will depend on the proportion of the population with immunity from natural infections or immunisation. Therefore, control rests on a population wide immunisation including children, which may or may not need to be repeated if new SARS-CoV-2 variants evolve that can escape immunity from either previous infections or immunisations. Preventing long term sequelae of COVID-19 also remains a priority.
